# Acute post-renal kidney graft dysfunction due to cytomegalovirus-positive nephrogenic adenoma—case report and review of the literature

**DOI:** 10.3389/fmed.2024.1394028

**Published:** 2024-05-29

**Authors:** Nicola Hosek, Matteo Montani, Laila-Yasmin Mani

**Affiliations:** ^1^Department of Medicine, Division of Nephrology and Dialysis, Kantonsspital Graubünden, Chur, Switzerland; ^2^Department of Nephrology and Hypertension, Inselspital, Bern University Hospital, University of Bern, Bern, Switzerland; ^3^Institute of Tissue Medicine and Pathology, University of Bern, Bern, Switzerland

**Keywords:** case report, nephrogenic adenoma, cytomegalovirus, kidney transplantation, allograft dysfunction, post-renal, infection, urologic complication

## Abstract

Tissue-invasive cytomegalovirus (CMV) disease represents a well-recognized complication after kidney transplantation. However, direct involvement of the urogenital tract and CMV-ureteritis occur less frequently. Nephrogenic adenomas are benign lesions of the urinary tract preferentially reported in kidney transplant recipients. We herein report a second case of a 33-year-old male kidney transplant recipient with acute post-renal allograft dysfunction due to CMV-positive ureteral nephrogenic adenoma. A causal connection might be suspected but remains to be proven.

## Introduction

Transplantation is the treatment of choice for patients with end-stage kidney disease (1). Given current highly effective immunosuppressive regimens, infectious complications represent a main cause for morbidity and mortality in patients after solid organ transplantation (2). Infections with cytomegalovirus (CMV) are among the most common opportunistic infections occurring in kidney transplant recipients and negatively affect transplant outcome (3). CMV-induced asymptomatic viremia and systemic disease are well recognized, as is tissue-invasive disease with classical involvement of the upper and lower gastrointestinal tract, the lungs or the liver (4). However, direct involvement of the kidney graft or the urogenital tract is rare.

Nephrogenic adenoma is a particular, uncommon benign lesion of the urinary tract with a wide range of histopathological characteristics mimicking malignant neoplasms (5). Nephrogenic adenomas mainly arise in the bladder while other locations in the urinary tract are less frequent (6). In kidney transplant recipients, the occurrence of nephrogenic adenoma in the bladder has been reported with incidences ranging from 0.53 to 4.3 per 100 transplants (7, 8). Despite various hypotheses, the underlying pathogenesis for the development of nephrogenic adenoma has not been completely elucidated to date.

Herein, we report a rare case of a kidney transplant recipient with acute post-renal allograft dysfunction due to CMV-positive ureteral nephrogenic adenoma and discuss a potential link between both conditions.

## Case description

A 33-year-old male of West-African descent with end-stage kidney disease due to hypertensive nephropathy received a kidney transplant from a deceased donor 6 years after initiating hemodialysis treatment. His past medical history was remarkable for hypertensive cardiopathy, chronic hepatitis B, and latent tuberculosis, for which treatment had been completed 2 years before transplantation.

Transplant allocation parameters included a kidney donor profile index of 4%, 0/8 HLA matches, and intermediate CMV and EBV-risk-constellations (donor+/recipient+). Transplant surgery was performed (left donor kidney into right iliac fossa) with a cold ischemia time of 11 h and a warm ischemia time of 27 min. The immunosuppressive regimen included basiliximab as induction therapy as well as cyclosporin A, mycophenolate mofetil (MMF) and prednisolone as maintenance therapy. According to local practice, a preemptive approach was followed using regular monitoring of CMV viremia without prophylactic antiviral therapy.

**Figure 1:**
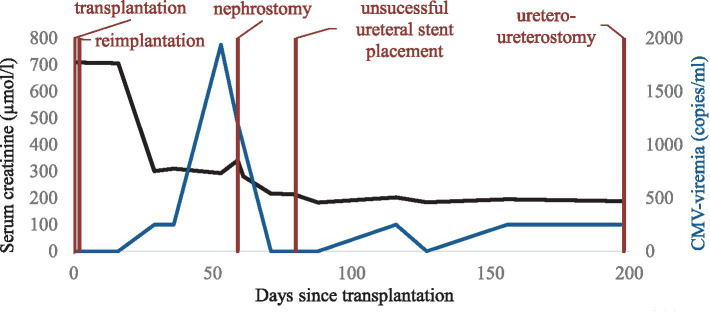
Timeline after kidney transplantation. CMV, cytomegalovirus.

The immediate postoperative course was complicated by delayed graft function requiring continued hemodialysis treatment. On postoperative day 2, magnetic resonance imaging (MRI) of the kidney graft showed subtotal stenosis of the transplant artery at the outflow of the right common iliac artery due to dissection of the right common iliac artery and kidney graft infarcts. Therefore, explantation, thromboendarterectomy of the right common iliac artery, ventral reconstruction of the common iliac artery using a pericard patch and re-implantation of the allograft was performed on the same day. Postoperative duplex ultrasound showed restored graft perfusion. Subsequently, graft function slowly recovered allowing discontinuation of hemodialysis therapy on postoperative day 13 and stabilization of graft function at a serum creatinine level of 270 µmol/l corresponding to an estimated glomerular filtration rate (eGFR) of 26 mL/min/1.73 m^2^ according to chronic kidney disease epidemiology (CKD-EPI) formula (Figure 1).

Two months post-transplant during a regular visit to the transplant outpatient clinic, increasing CMV viremia of 1940 copies/ml was detected after low grade viremia at <500 copies/ml had been weekly monitored since a month post-transplant. At this time, valganciclovir at therapeutic dosage was started. Only 6 days later, a rise in serum creatinine to 341 μmoL/L was noted as well as new-onset microhematuria that had been retrospectively present since the preceding week. Duplex ultrasound of the kidney graft newly revealed hydronephrosis grade III. Consequently, urgent percutaneous nephrostomy was placed leading to a prompt fall in serum creatinine. Three weeks after treatment start, CMV viremia was undetectable and valganciclovir was stopped. Due to this satisfying response to valganciclovir, MMF was maintained at the same dose of 2 g per day (Figure 1). BK viremia was undetectable throughout follow-up.

**Figure 2:**
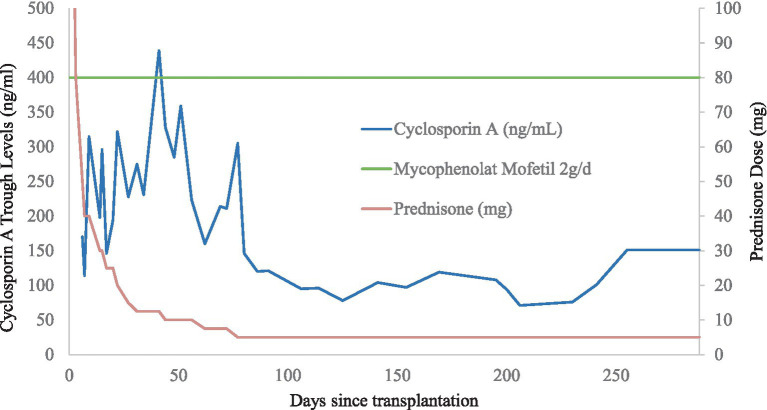
Immunosuppressive regimen and cyclosporin A trough levels after transplantation.

Three months after nephrostomy placement, further urologic work-up by antegrade pyelography was performed revealing a stenotic ureteral lesion in proximity to the bladder. However, an attempt to insert a ureteral stent during the same session was unsuccessful. Therefore, definitive surgical treatment was undertaken consisting of secondary uretero-ureterostomy with the ipsilateral native ureter. Subsequently, the nephrostomy could be removed, and the serum creatinine remained stable at 193 μmoL/L (Figure 2).

**Figure 3:**
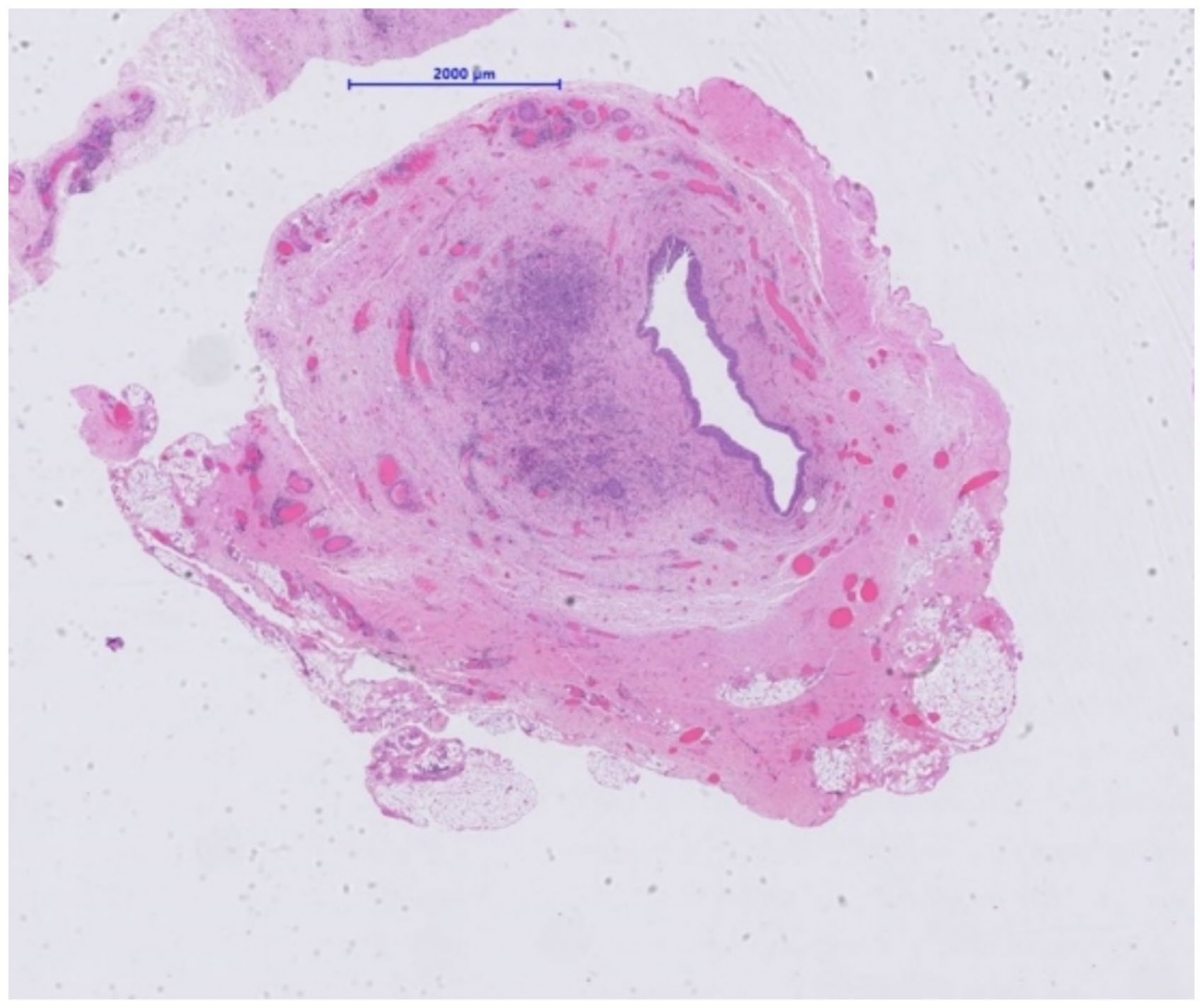
Resected ureter segment. Narrowed lumen in transplant ureter due to the presence of a cellular proliferation (H&E, x 10).

**Figure 4:**
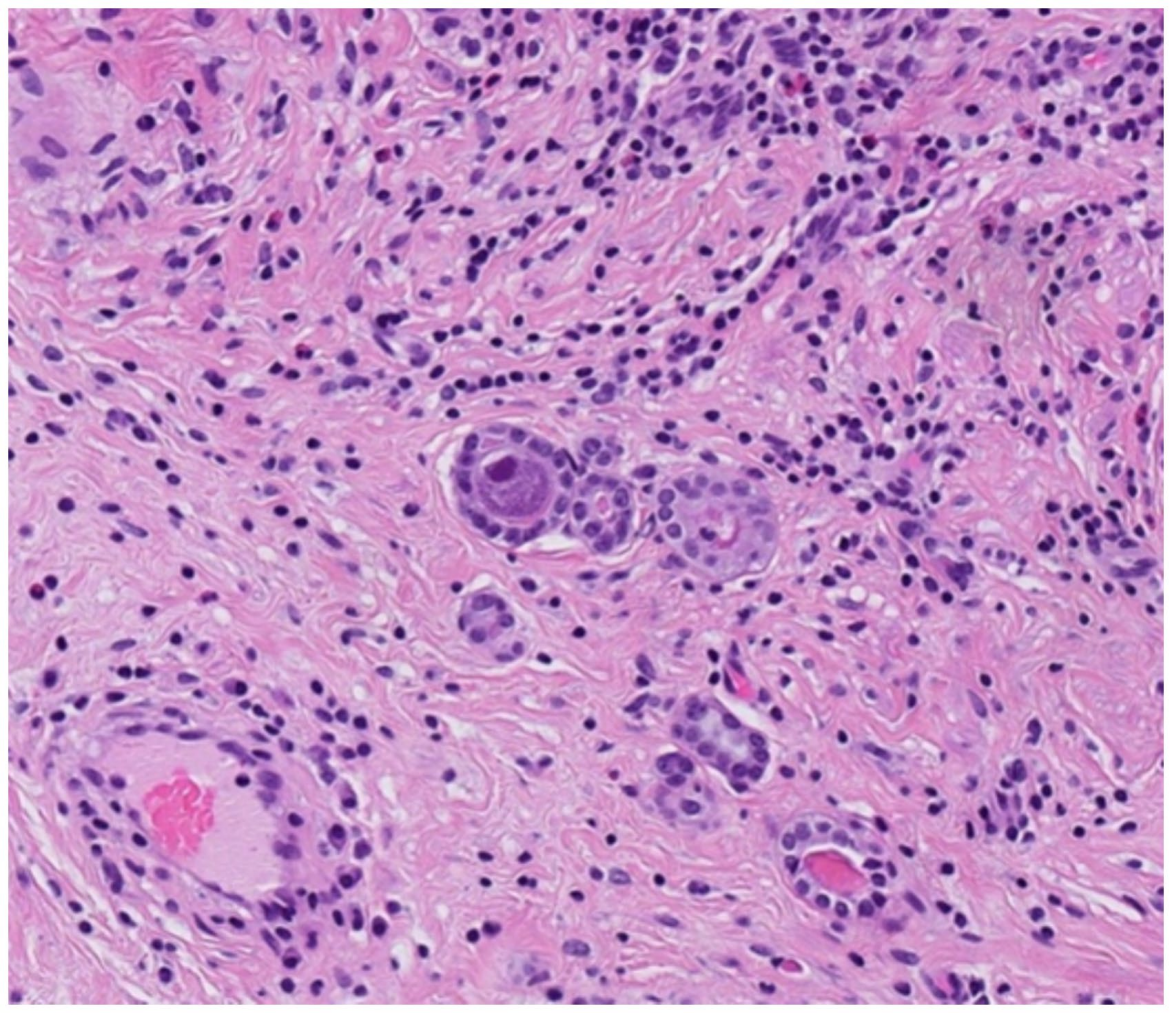
Resected ureter segment. Microglandular proliferation of a nephrogenic adenoma with typical cytopathic appearance of a CMV-infected cell (H&E, x 200).

**Figure 5:**
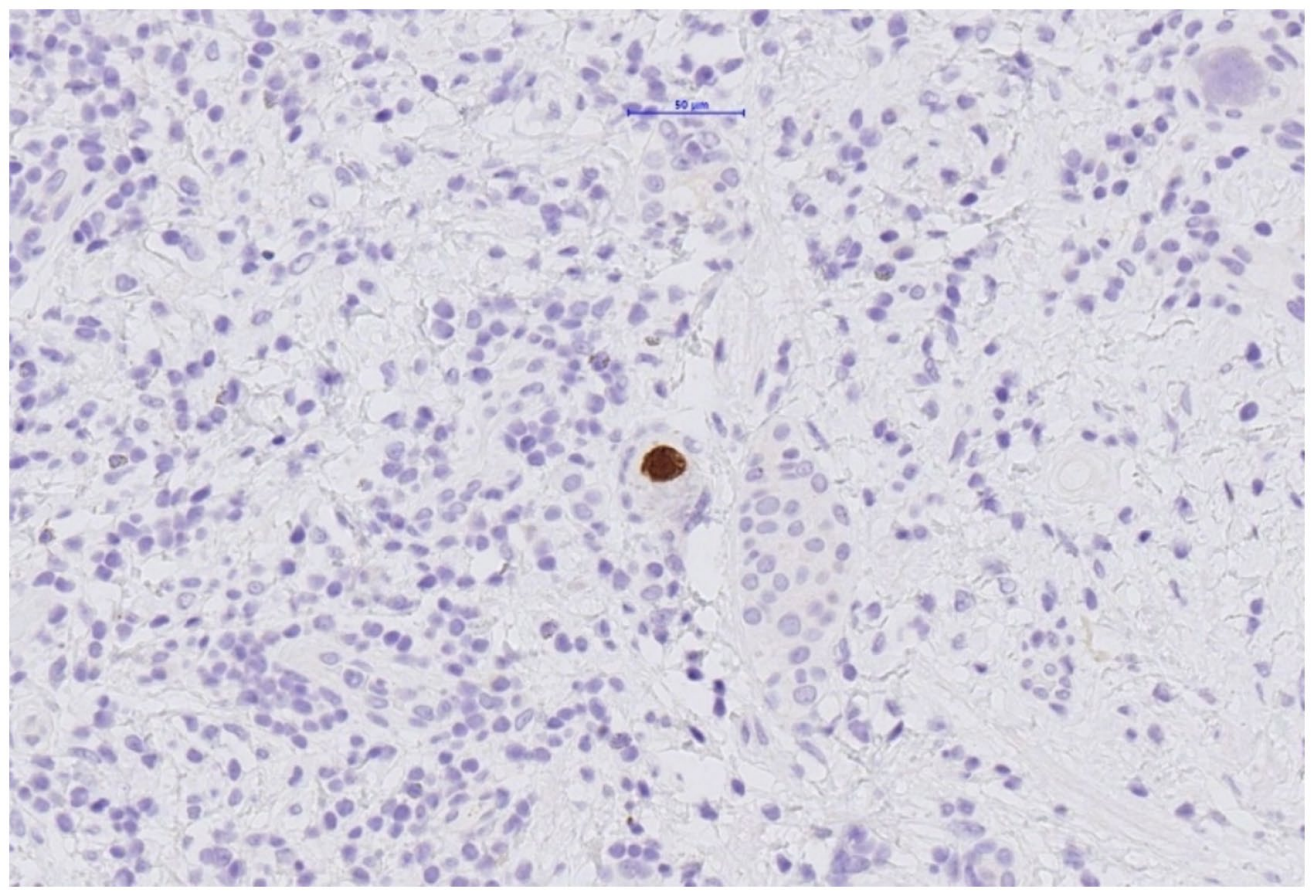
Resected ureter segment. Cells within nephrogenic adenoma with positive immunohistochemic staining for CMV.

The histological examination of the resected transplant ureter showed presence of a PAX8-positive cell proliferation with surrounding fibrosis (Figure 3) consistent with nephrogenic adenoma. There were no signs of malignancy. Surprisingly, several cells showed cytopathic changes characteristic for CMV (Figure 4) and immunohistochemistry was positive for cytomegalovirus (CH2- and DDG-antibodies, Dako, dilution 1:400, pre-treatment H2 30 95; Figure 5). In the simultaneously taken kidney graft sample, signs of acute tubular injury without further anomalies were seen; however, tissue sampling was limited. CMV immunohistochemistry and SV40 staining, as BK-virus marker, were negative.

In the presence of CMV-positive nephrogenic adenoma, another course of valganciclovir at therapeutic dosage was introduced for 6 weeks. Immunosuppressive therapy was maintained unchanged (Figure 1). The following clinical course was unremarkable with a serum creatinine value measured at 136 µmol/l at last follow-up and disappearance of hematuria after the last urologic intervention.

## Discussion

We present the case of a kidney transplant recipient with acute post-renal kidney graft dysfunction due to CMV-positive nephrogenic adenoma of the ureter. To the best of our knowledge, this is the second described case of ureteral nephrogenic adenoma with CMV superinfection to date.

Acute kidney graft dysfunction has been estimated to occur with an incidence of 21% in the first 6 months after kidney transplantation and adversely affects patient and transplant outcomes (9, 10). Causes for acute kidney graft dysfunction include transplant-specific etiologies such as acute rejection, BK virus nephropathy and calcineurin inhibitor toxicity in addition to classical causes of acute kidney injury. In a single center cohort of 326 kidney transplant recipients, urinary tract obstruction accounted for 10% of cases of acute kidney injury in the early post-transplant period (9). Both CMV ureteritis and nephrogenic adenoma represent possible, albeit infrequent causes for post-renal acute kidney graft dysfunction.

CMV ureteritis is a rare manifestation of CMV-related tissue-invasive disease that has been increasingly recognized in kidney transplant recipients during the last decades and been linked to the progressive use of mycophenolate in the transplant setting (11–15). Its main manifestations include mild fever, urinary obstruction and kidney impairment. Risk factors for the development of CMV ureteritis are acute allograft rejection, the use of depleting immunosuppression or MMF as well as the absence of prophylactic antiviral therapy (11, 12). In our patient, use of an MMF-based immunosuppression and lack of antiviral prophylaxis following the preemptive therapy approach might have favored the occurrence of CMV-associated tissue-invasive disease.

Nephrogenic adenoma of the urinary tract may present with various symptoms. According to a single center retrospective analysis of 32 cases of nephrogenic adenoma, symptoms were present in 72% of patients including hematuria, urinary symptoms or incontinence, flank pain and hydronephrosis (6). In our patient, new-onset microhematuria was retrospectively noted 1 week before acute worsening of graft function together with the finding of hydronephrosis. There were no urinary symptoms nor painful graft site.

Until now, the pathogenesis of the development of nephrogenic adenoma remains incompletely understood. Several hypotheses have been put forward including the development from remnant mesonephric tissue, the development as metaplastic response to local trauma, irritation, inflammation or immunosuppression as well as the development from shed, secondarily implanted renal tubular cells (16). Indeed, in a landmark study in 24 kidney transplant recipients, bladder nephrogenic adenoma has been shown to derive from the kidney graft (i.e., donor) using fluorescence *in situ* hybridization studies of sex chromosomes (17). However, controversial data exist outside the transplant setting (16).

In our patient, nephrogenic adenoma of the ureter was found to be CMV-positive. We are aware of four previously reported cases of CMV-positive nephrogenic adenomas in kidney transplant recipients (18–21); while three of them affected the bladder, only one case involving the transplant ureter has been described so far (Table 1). Most of the cases were diagnosed within 1 year post-transplant, all of them by histological analysis. In the majority of general cases of nephrogenic adenoma in kidney transplant recipients published so far, CMV testing has not been reported (Table 2) (7, 8, 17, 22–36). On one hand it may be speculated whether CMV-induced local inflammation may have predisposed to the development of nephrogenic adenoma. CMV encodes several proteins inhibiting the assembly and trafficking of cellular proteins, which participate in immune recognition (e.g., major histocompatibility complex 1 and major histocompatibility complex 2). Consequently, CMV hides infected cells from adaptive immunity (37). This immune evasive capability not only helps CMV to persist within its host cells, but may further may predispose to the formation of metaplasia such as nephrogenic adenoma. In addition, sequential surgical procedures may have played a causative role in our case. Thus, the explantation and reimplantation of the allograft due to the artery dissection may have led to substantial shedding of tubular epithelial cells into the ureter and bladder of our patient finally leading to the formation of nephrogenic adenoma (17). On the other hand, nephrogenic adenoma *per se* may have favored CMV reactivation. Indeed, CMV reactivation secondary to inflammatory stimuli has been suggested previously (38). Interestingly, nephrogenic adenoma of the bladder positive for BK polyomavirus has similarly been reported in a kidney transplant recipient (34). According to the histological findings, the authors suggested BK virus contributed to cell atypia, but was not a causative factor for the development of nephrogenic adenoma.

**Table 1:** Reported cases of nephrogenic adenoma with CMV-infection after kidney transplantation.

Author	Time after kidney transplantation	Symptoms	Location	Detection of CMV	Therapy
Beaudry 1983 (18)	5.33 years	Gross hematuria	Over 50% of bladder surface including the site of the reimplanted ureter	Biopsy and serum	Withdrawal of azathioprine
Buzelin 1988 (19)	2.4 years	Gross hematuria, dysuria	Bladder	Biopsy	Resection
Redman 2000 (20)	1 year	Vesical calculi	Bladder next to the ureteroneo-cystostomy	Biopsy	Resection
Hung 2001 (21)	3 months	Ureteral obstruction, gross hematuria	Ureter	Biopsy	Resection, Valganciclovir, withdrawal of azathioprine
Our case	2 months	Ureteral obstruction, hematuria	Ureter	Biopsy and serum	Valganciclovir and resection

**Table 2:** Previous cases of nephrogenic adenoma after kidney transplantation.

Year	Author	Number of Cases	Supposed precipitating factors	CMV testing	Total
1975	Gordon et al. (22)	1	Impaired immunologic surveillance	Not reported	1
1982	Behesti et al. (23)	1	Renal transplantation, UTI	Not reported	2
1988	Gonzalez et al. (24)	8	Renal transplantation	Not reported	10
1992	Zeidan et al. (25)	2	Renal transplantation, transurethral resection of the prostate	Not reported	12
1995	Colombo et al. (26)	5	Mechanical trauma and recurrent UTI	3 with postoperative systemic CMV infection, 2–4 years before nephrogenic adenoma	17
1996	Fournier et al. (8)	9	Ureterovesical anastomosis, chronic prostatitis, vesicorenal reflux, cyclophosphamide, condyloma	No viroid inclusions in biopsy	26
1997	Tse et al. (27)	7	Immunosuppression, ureterovesical anastomosis, recurrent UTI, changes of JJ stents	Not reported	33
1998	Banyai-Falger et al. (7)	7	Recurrent UTI, surgical procedure of renal transplantation	2 with CMV disease, unrelated to nephrogenic adenoma	40
1998	Pycha et al. (28)	12	Renal transplantation, inflammation	Not reported	52
2002	Whang et al. (29)	1	Kidney-pancreas transplantation with drainage of the pancreas into the bladder	Not reported	53
2002	Mazal et al. (17)	29	Shedding of renal tubular cells due to trauma, infection and/or immunosuppression	Not reported	82
2008	Kim et al. (30)	1	UTI and bladder stones	Not reported	83
2009	Ladenheim et al. (31)	1	Renal transplantation	Not reported	84
2013	Voss et al. (32)	1	Renal transplantation	Not reported	85
2014	Kuzaka et al. (33)	2	Renal transplantation, immunosuppression, reoperation	Not reported	87
2015	Alexiev et al. (34)	1	BK Virus infection	Not reported	88
2017	North et al. (35)	1	Recurrent UTI	Not reported	89
2020	Kahn et al. (36)	1	Surgery	Not reported	90

CMV, cytomegalovirus; UTI, urinary tract infection; JJ, double J.

Currently supported preventive strategies for CMV in kidney transplant recipients include prophylactic and preemptive therapy approaches (39). Preemptive CMV therapy includes regular monitoring of CMV viremia and start of antiviral therapy in case of viral replication at pre-specified levels. In patients with high-risk constellation (donor +/ recipient -) and after induction with depleting agents, a prophylactic approach may be chosen (40). However, the diagnosis of CMV-related tissue-invasive disease requires detection of CMV in the tissue by histology (cytopathic changes) or immunohistochemistry (39). In addition, CMV-related tissue-invasive disease may occur in the absence of CMV viremia as has also been reported for CMV ureteritis (14). Fortunately, in our patient, local symptoms were accompanied by a simultaneous rise in CMV viremia leading to prompt start of antiviral treatment, although being stopped after viremia was undetectable. However, the clinical course of our patient might advocate for a lower viremia threshold for instauration of antiviral therapy. Indeed, during the month preceding the acute rise in CMV viremia, low-grade viremia (< 500 copies/ml) had been present.

Management of the patient with ureteral nephrogenic adenoma reported by Hung et al. included resection of the lesion and pyeloplasty, intravenous ganciclovir treatment for 2 weeks and withdrawal of the antimetabolite azathioprin (21). The optimal duration of antiviral treatment for CMV-related tissue-invasive disease is not known. However, longer therapy courses are generally admitted in these cases (39). Therapy of nephrogenic adenoma usually involves endoscopic resection of the lesion with variable reported recurrence rates (6, 41). In our patient, valganciclovir treatment was re-started after diagnosis of CMV-related tissue-invasive disease for a total of 6 weeks without concomitant change in immunosuppression. Given the shortness of the lesion-free transplant ureter, surgical reconstruction after ureter resection involved proximal uretero-ureterostomie between transplanted and patient ureter.

In conclusion, CMV-ureteritis and nephrogenic adenomas are rare causes for acute post-renal kidney graft dysfunction. Diagnosis of tissue-invasive CMV disease requires histological evidence of CMV at the affected site. To the best of our knowledge, this is the second reported case of ureteral nephrogenic adenoma with CMV superinfection in a kidney transplant recipient. A causal link might be suspected but remains to be proven (42, 43).

## Data availability statement

The original contributions presented in the study are included in the article/supplementary material, further inquiries can be directed to the corresponding author.

## Ethics statement

Written informed consent was obtained from the individual(s) for the publication of any potentially identifiable images or data included in this article.

## Author contributions

NH: Investigation, Visualization, Writing – original draft, Writing – review & editing. MM: Resources, Writing – review & editing. L-YM: Supervision, Investigation, Writing – original draft, Writing – review & editing.
